# Supplementary material for: Comparative multiomics analysis of cell physiological state after culture in a basket bioreactor
Source: Sci Rep. 2022 Nov 23;12:20161. doi: 10.1038/s41598-022-24687-4 (PMC9686226; doi:10.1038/s41598-022-24687-4)
Supplement: Supplementary file 1 — Supplementary Information 1. [file 41598_2022_24687_MOESM1_ESM.zip › raw data/Transcriptomic raw data/6.Enrichment/1.GO/TE1vsTC1/GSEA/TE1/ORGANIC_ACID_TRANSMEMBRANE_TRANSPORTER_ACTIVITY/ORGANIC_ACID_TRANSMEMBRANE_TRANSPORTER_ACTIVITY(GO_0005342).html]

Details for gene set ORGANIC\_ACID\_TRANSMEMBRANE\_TRANSPORTER\_ACTIVITY(GO:0005342)[GSEA]


|  || Dataset | readcount.sample |
| Phenotype | sample.cls |
| Upregulated in class | TE1 |
| GeneSet | ORGANIC\_ACID\_TRANSMEMBRANE\_TRANSPORTER\_ACTIVITY(GO:0005342) |
| Enrichment Score (ES) | 0.7133003 |
| Normalized Enrichment Score (NES) | 2.0415833 |
| Nominal p-value | 0.0 |
| FDR q-value | 0.1949335 |
| FWER p-Value | 0.32 |
Table: GSEA Results Summary

  

Fig 1: Enrichment plot: ORGANIC\_ACID\_TRANSMEMBRANE\_TRANSPORTER\_ACTIVITY(GO:0005342)
  
Profile of the Running ES Score & Positions of GeneSet Members on the Rank Ordered List

  

| PROBE | DESCRIPTION   (from dataset) | GENE SYMBOL | GENE\_TITLE | RANK IN GENE LIST | RANK METRIC SCORE | RUNNING ES | CORE ENRICHMENT || 1 | 103215389 | SLC7A2 |  |  | 643 | 9.149 | 0.2885 | Yes |
| 2 | 103233435 | SLC7A5 |  |  | 1570 | 2.396 | 0.3303 | Yes |
| 3 | 103220154 | SLC1A4 |  |  | 1972 | 2.098 | 0.3851 | Yes |
| 4 | 103236270 | SLC7A11 |  |  | 2067 | 2.038 | 0.4516 | Yes |
| 5 | 103233242 | SLC7A6 |  |  | 2542 | 1.840 | 0.4942 | Yes |
| 6 | 103231465 | SLC7A8 |  |  | 2612 | 1.817 | 0.5541 | Yes |
| 7 | 103237078 | SLC7A13 |  |  | 2716 | 1.789 | 0.6115 | Yes |
| 8 | 103235521 | SLC1A5 |  |  | 2951 | 1.718 | 0.6607 | Yes |
| 9 | 103214212 | SLC7A1 |  |  | 3805 | 1.554 | 0.6765 | Yes |
| 10 | 103231442 | SLC7A7 |  |  | 4757 | 1.428 | 0.6835 | Yes |
| 11 | 103215141 | SLC1A3 |  |  | 5165 | 1.383 | 0.7133 | Yes |
| 12 | 103219505 | SLC1A1 |  |  | 11452 | 0.929 | 0.4647 | No |
| 13 | 103234451 | SLC7A9 |  |  | 18010 | 0.489 | 0.1888 | No |
| 14 | 103237679 | SLC1A2 |  |  | 20406 | 0.210 | 0.0891 | No |
| 15 | 103234456 | LOC103234456 |  |  | 22378 | 0.000 | 0.0011 | No |
Table: GSEA details
[plain text format]

  

Fig 2: ORGANIC\_ACID\_TRANSMEMBRANE\_TRANSPORTER\_ACTIVITY(GO:0005342)
  
Blue-Pink O' Gram in the Space of the Analyzed GeneSet

  

Fig 3: ORGANIC\_ACID\_TRANSMEMBRANE\_TRANSPORTER\_ACTIVITY(GO:0005342): Random ES distribution
  
Gene set null distribution of ES for
 **ORGANIC\_ACID\_TRANSMEMBRANE\_TRANSPORTER\_ACTIVITY(GO:0005342)**
